# A functionalized graphene oxide with improved cytocompatibility for stimuli-responsive co-delivery of curcumin and doxorubicin in cancer treatment

**DOI:** 10.1038/s41598-022-05793-9

**Published:** 2022-02-04

**Authors:** Fatemeh Yaghoubi, Najmeh Sadat Hosseini Motlagh, Seyed Morteza Naghib, Fateme Haghiralsadat, Hossein Zarei Jaliani, Ali Moradi

**Affiliations:** 1 Department of Clinical Biochemistry, School of Medicine, Shahid Sadoughi University of Medical Sciences, Yazd, Iran; 2 Herbal Medicine Research Center, Faculty of Pharmacy, Shahid Sadoughi University of Medical Sciences, Yazd, Iran; 3 Department of Biomedical Engineering, Meybod University, Meybod, Iran; 4 Nanotechnology Department, School of Advanced Technologies, Iran University of Science and Technology (IUST), P.O. Box 16846-13114, Tehran, Iran; 5 Department of Advanced Medical Sciences and Technologies, School of Paramedicine, Shahid Sadoughi University of Medical Sciences, Yazd, Iran; 6 Medical Nanotechnology & Tissue Engineering Research Center, Department of Advanced Medical Sciences and Technologies, Yazd Reproductive Sciences Institute, Shahid Sadoughi University of Medical Sciences, Yazd, Iran; 7 Department of Medical Biotechnology, School of Medicine, Shahid Sadoughi University of Medical Sciences, Yazd, Iran

**Keywords:** Cancer therapy, Graphene, Nanobiotechnology, Nanomedicine, Nanoscale devices, Nanoscale materials

## Abstract

Nowadays, the usage of nanoparticles in various fields such as drug delivery, attracts the attention of many researchers in the treatment of cancers. Graphene oxide (GO) is one of the novel drug delivery systems which is used broadly owing to its unique features. In this survey, doxorubicin (DOX) was accompanied by natural medicine, curcumin (CUR), to diminish its side effects and enhance its efficiency. Cytotoxicity assay in human gastric cancer (AGS), prostate cancer (PC3), and ovarian cancer (A2780), was evaluated. Also, the uptake of DOX and CUR into cells, was assessed using a fluorescence microscope. Moreover, real-time PCR was applied for the evaluation of the expression of RB1 and CDK2 genes, which were involved in the cell cycle. In both separate and simultaneous forms, DOX and CUR were loaded with high efficiency and the release behavior of both drugs was pH-sensitive. The higher release rate was attained at pH 5.5 and 42 °C for DOX (80.23%) and CUR (13.06), respectively. The intensity of fluorescence in the free form of the drugs, was higher than the loaded form. In the same concentration, the free form of CUR and DOX were more toxic than the loaded form in all cell lines. Also, free drugs showed more impact on the expression of RB1 and CDK2 genes. Co-delivery of CUR and DOX into the mentioned cell lines, was more effective than the free form of CUR and DOX due to its lower toxicity to normal cells.

## Introduction

Cancer is one of the main causes of death in the world^1–3^. The most common strategies for the treatment of cancer are surgery, radiotherapy, and chemotherapy^4–6^. Although the effectiveness of chemotherapy has been confirmed in the past three decades, the major obstacle is its toxicity and side effects on normal cells^7^. Other obstacles in the treatment of cancer, are extensive heterogeneity of cancer cells and drug resistance that lead to the efflux of anticancer drugs from tumor cells, as well as in chemo-resistance, the expression of the anti-apoptotic proteins is increased^8^. Heterogeneity is originated from epigenetic differences and the DNA instability of tumor cells, which in turn, can lead to different responses to therapy. As a result, the combination treatment can be used as a more effective strategy than single-agent therapy, owing to targeting multiple pathways of cancer cells^9^. Many researchers have eagerly tried to use the nano-carriers, due to their great advantages, such as the reduction in systemic toxicity of the loaded drugs, and the capability of carrying multiple drugs simultaneously^10^. DOX is a chemotherapeutic agent which inhibits tumor growth by blocking the function of topoisomerase, resulting in DNA double helical breaks leading to activation of cell cycle arrest or apoptosis^11^. CUR is a herbal medicine that possesses anti-oxidant, anti-inflammatory, and anticancerous properties^12^. In fact, CUR impacts on the multiple cell signaling pathways, including proliferation (EGFR, HER-2, and AP-1), apoptosis (activation of caspases and downregulation of antiapoptotic gene products), angiogenesis (VEGF) and inflammation (NF-kB, IL-6, IL-1, TNF, COX-2, and 5-LOX)^13^. RB1 gene regulates the transition from G1/G0- to S-phase in the cell cycle, and activates cell cycle arrest in response to DNA damage^14^.

Nanoscaled materials and nanostructures are increasing considerable attentions for biomedical applications such as tumor theranosis^15^ and combination therapy^16,17^. Nano-designed materials assist combining the diagnosis with therapy, which are the critical components of a modified approach to deal with the malignancy^18^. Nanoparticles as drug carriers, not only transport therapeutic molecules but also transfer the hydrophobic agents^19–21^. There are some limitations in the administration of CUR into cells, including low absorption and solubility, rapid metabolism and rapid systemic elimination of CUR in the body^19,22^. Therefore, various nano-carriers have been studied in last decades. Among nanoscaled materials, graphene and its derivatives are the promising nanocarriers that have several benefits over others^23^, because of their exceptional characteristics including high surface area, mechanical and chemical stability, 2D planar structure, good cytocompatibility and excellent conductivity^24^. The planar structure of graphene makes it a suitable factor for the high loading of different substances, such as biomolecules and metals. On the other hand, GO can deliver small drug molecules (such as anticancer and antibacterial agents) and macromolecules as well as its bipolar groups (hydrophilic and hydrophobic) allow it to carry both hydrophilic and hydrophobic substances. Altogether these excellent properties along with their small size and high biocompatibility, make GO a promising candidate for medical and biological applications^25^. The functionalization of GO with oxygenated groups such as carboxyl, increases its biocompatibility and solubility^26^. Some studies have shown the higher biocompatibility, safety and efficacy of drug loaded in GO-COOH rather than GO^27,28^.

Several studies have been conducted in this field. One study assayed the cytotoxicity of graphene oxide and graphene oxide loaded with doxorubicin on human multiple myeloma cells, and found that the cell proliferation was inhibited significantly by GO/DOX compared to pure DOX^29^. Another study represented that the release of CUR from PEGylated GO, was depended on pH which was increased in the basic environment^30^. Also, co-loading of CUR and paclitaxel on polymer-functionalized reduced GO, was performed in another study, which the results showed a synergistic treatment and a highly potent nano-carrier towards the breast, MDA-MB-231, lung and A549 cancer cells^31^.

The present study aimed to improve the cytotoxicity effect of CUR by co-delivery of DOX loaded on GO-COOH. In fact, the cytotoxicity effect of free CUR, free DOX, GO, GO-CUR, GO-DOX and GO-CUR-DOX as well as the uptake of the mentioned drugs and the expression rate of RB1 and CDK2 genes in AGS, A2780, PC3 and HFF cell lines, were studied.

## Materials

DOX was obtained from Ebewe Pharma (Austria). CUR (purity > 65%), DMSO (dimethyl sulfoxide), MTT (3–(4, 5-dimethylthiazol-2-yl)-2, 5-diphenyl tetrazolium bromide), dialysis bag (MW¼ 12 kDa) and PBS tablets were supplied from Sigma-Aldrich (St Louis, MO). DIL Stain (1, 10-Dioctadecyl-3,3,30,30-Tetramethylindocarbocyanine Perchlorate) and DAPI (40, 6-diamidino2-phenylindole) were produced from Thermo Fisher Scientific (Waltham, MA). GO was purchased from GrapheneX. HCL 37% and Ethanol were provided by Merck (New Jersey).

## Methods

### Morphological assessment and zeta potential

Zeta potential was assessed using Brookhaven Corp Instruments (Holtsville, NY). Also, the structure of GO-COOH was evaluated by scanning electron microscope (SEM). For this purpose, a thin layer of film was created after pouring 5 µl of suspension on the glass plate. Then, after coating the sample with a gold layer, the images were recorded by scanning electron microscopy (model EM3200, KYKY, China).

### Preparation of GO-COOH

For the GO carboxylation, GO (2 mg/ml) was sonicated for 1 h. Then, 72 mg of NaOH was added and stirred at room temperature for 4 h. Then, 0.4 ml of HCl (37% v/v) was added to the solution and washed several times with deionized water for removing the salts. As a result, the GO-COOH compound was prepared^32–34^.

### Drug loading on GO-COOH

For preparing GO-COOH-DOX and GO-COOH-CUR, DOX (0.5 mg/ml solution in PBS) and CUR (0.5 mg/ml dissolved in ethanol) were mixed with GO-COOH (0.5 mg/ml), respectively and stirred overnight at room temperature. In the combination form, DOX (0.5 mg/ml) and CUR (0.5 mg/ml) were mixed with GO-COOH (0.5 mg/ml) at the same ratio (1:1). Centrifuging at 15,000 g for 10 min, is employed for removing unbounded drug which its concentration is calculated via measuring the absorption of DOX and CUR at 480 nm and 430 nm, respectively by a UV–Vis spectrophotometer (Epoch Box 998 America). Afterward, the following equation was used for calculating entrapment efficiency (EE%):

$${\text{EE}}\% = \left( {{\text{Loaded}}\;{\text{ drug}}\;{\text{ on}}\;{\text{GO - COOH}}\;\left( {{\text{mg}}\;{\text{ ml}}^{{ - {1}}} } \right)/{\text{Total }}\;{\text{drug}}\left( {{\text{mg}}\;{\text{ml}}^{{ - {1}}} } \right)} \right) \times {1}00$$

### Release assay

A 12 kDa cut-off dialysis tube was applied for the release assay of DOX and CUR from GO-COOH, which was immersed in PBS, while stirred for 72 h at pH 7.4 and 5.5 at 37 °C and 42 °C. The dialysis solution over the dialysis tube was gathered (0.5 ml) at different time intervals and substituted with fresh PBS (0.5 ml). Then its absorbance was measured by the UV–Vis spectrophotometer. The percentage of release in different times, was calculated based on the total loaded drug concentration.

### Cell culture assay

The cell lines of human gastric cancer (AGS), prostate cancer (PC3) and ovarian cancer (A2780) were purchased from Pasteur Institute (Tehran, Iran) and human foreskin fibroblast (HFF) cell line, a normal cell line, was supplied from Stem Cell Biology Research Center (Yazd, Iran). All cell lines were cultured under standard conditions (37 °C and 5% CO_2_) in DMEM medium (Gibco, Grand Island), encompassing 10% FBS (fetal bovine serum) (Gibco Grand Island) and penicillin–streptomycin (Gibco, Grand Island).

### In vitro cellular uptake

The distribution of the free form of DOX and CUR in combination with GO-COOH into the cells, was detected via fluorescence intensity. First, all cell lines (1.5 × 10^5^ per well) were cultured in a 6-well plate, and were treated with free DOX, free CUR, free CUR-DOX, GO-COOH-CUR, GO-COOH-DOX, and GO-COOH-CUR-DOX. After incubation for 3 h, all cells were washed with PBS (pH 7.4), and 95% ethanol solution was used as a fixative. At the last stage, after staining cells with DAPI (1 mg/ml), images were captured by fluorescence microscopy (Olympus, Japan).

### Cytotoxicity study

IC50 doses of blank GO-COOH (62.5, 125, 250, 500 and 1000 µM), free DOX and GO-DOX (0.31, 0.625, 1.25, 2.5, 5 and 10 µg/ml), free CUR and GO-CUR (3.9, 7.8, 15.6, 31.25, 62.5 and 125 µg/ml) were calculated by MTT assay after 48 h, Also, co-administration of these drugs on GO-COOH, was performed at different concentrations (4.1, 8.125, 16.25, 32.5, 65, and 130 µg/ml). After removing the content of wells, incubation with 10 µl of MTT (5 mg/ml) and 90 µl of medium was executed for 3 h. In the next step, DMSO was used for dissolving the formazan crystals. Finally, the absorbance of the samples was evaluated through an EPOCH spectrophotometer at 570 nm (Bio-Tek, Winooski).

### Real time PCR

Real time PCR was used for assessment of the expression of RB1 and CDK2 genes in the cell cycle. For this purpose, 150,000 cells (AGS, A2780, PC3 and HFF) from each cell line per well, were seeded in six-well plates. After 24 h, the cells were treated with free CUR, free DOX, GO, GO-CUR, GO-DOX and GO-CUR-DOX. Then, after 48 h and removing the medium, cells were washed with 1 ml PBS. Total RNA was extracted using RNX-Plus extraction kit which was followed by Parstous cDNA synthesis Kit. Then, using specific primers for RB1 and CDK2 genes and Yektatajhiz master mix, quantitative real-time PCR was conducted. Beta-actin was applied as a housekeeping gene. Ultimately, the rate expressions of RB1 and CDK2 were evaluated through 2^-ΔΔCT^ method. Beta-actin was used as s house holding gene.

### Statistical analysis

Data were analyzed using GraphPad Prism version 6 (GraphPad, San Diego, CA) that was expressed as the mean ± standard deviation (SD). One-way analysis of variance (ANOVA) and Tukey's multiple comparison test were applied for the measurement of statistical difference (P-value < 0.05).

## Results and discussion

### Characterization of nano-formulation

#### Uv–visible analysis and morphological characterization

**Figure 1:**
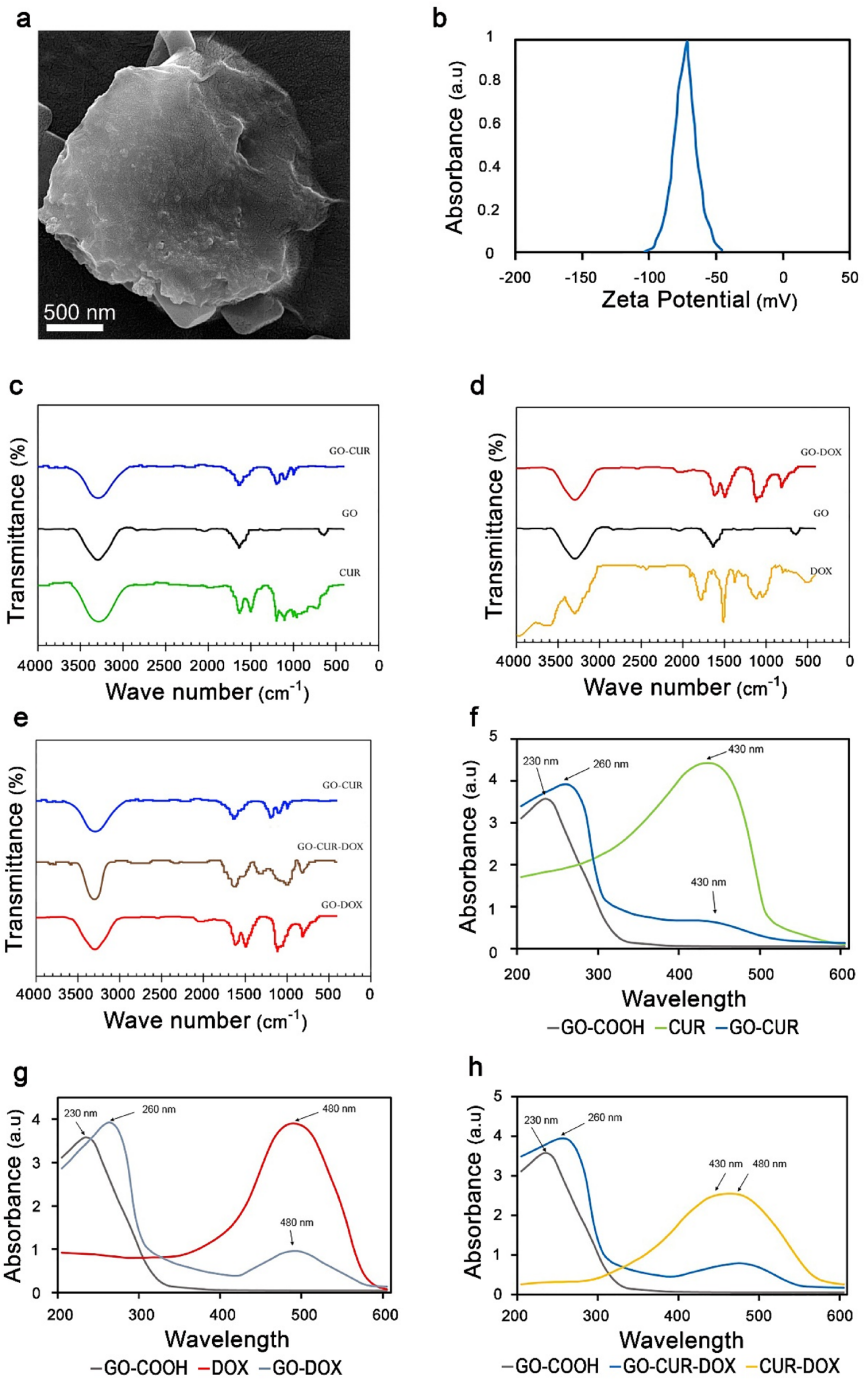
(**a**) SEM image of GO-COOH. (**b**) Zeta potential of GO-COOH. (**c**–**e**) FTIR of free drugs and GO-COOH-loaded drugs. UV–Vis spectra of (**f**) CUR, GO-COOH, GO-CUR (**g**) DOX, GO-COOH, GO-DOX (**h**) CUR-DOX, GO-COOH, GO-CUR-DOX.

The SEM photographs of GO-COOH are depicted in Fig. 1a which show the broad surface and thin layer of GO-COOH. Cellular interactions in the physiological system, depend on the surface charge, which dynamic light scattering (DLS) was used to determine the zeta potential of GO-COOH. In fact, zeta potential measures the attraction or repulsion between the particles^35^. Stabilization of the particles can be determined by the amount of zeta potential. The higher value of zeta potential (positive or negative) is related to greater stability. However, in the lower value of zeta potential, aggregation defeats the dispersion^36,37^. Figure 1b displays the zeta potential of GO-COOH which its negative charge (− 70.0 mV) indicates the presence of COOH in the surface. Figure 1c–e displays the FTIR spectra of the nanomaterials. OH vibration of GO-COOH is depicted as a broadband at ∼ 3301 cm^−1^ while a functional group of GO-COOH (C=O) is shown at ∼ 1631 cm^−1^^33^. Figure 1d shows the related picks of DOX at 1115 cm^−1^ and 813 cm^−1^, corresponding to stretching bands of C-O-CH_3_. Also, GO-DOX displays two principal picks of GO functional groups, 3301 and 1631 cm^−1^, and correspondence picks of DOX, 1110 and 814 cm^−1^, which presented successful loading of DOX onto GO. Cur exhibits the peaks at 3300 cm^−1^, 1450–1640 cm^−1^ and 1000–1300 cm^−1^ that belong to O–H, C=C and C–O–C, respectively^38–41^. GO-CUR displays the peaks at 3300 cm^−1^, 1640 cm^−1^ and 1000–1300 cm^−1^, confirming the loading of Cur.

The UV–Vis spectra of free CUR, free DOX, free CUR-DOX, GO-COOH, GO-CUR, GO-DOX and GO-CUR-DOX are depicted in Fig. 1f–h. The spectrum of GO-COOH is identified by a single peak at 230 nm, that is attributed to the molecular transition of π → π* for C–C aromatic rings^42^. Moreover, the maximum absorbance of free CUR and free DOX are shown at 430 and 480 nm, respectively while in the loaded form, the red shift of GO-COOH (260 nm) is added to the specific peak of every drug^30,43^.

### Loading assay

Achieving high concentrations of loaded CUR and DOX on GO-COOH, was our main goal. For this purpose, the maximum loading concentrations of each drug was measured. Then, simultaneous loading of CUR and DOX was conducted at the same concentration of maximum drug loading. pH plays an important role in the loading of drugs on GO-COOH. In order to achieve maximum loading, it is necessary that the pH of drugs must be close to the pH of GO-COOH (5.5–6), otherwise, GO-COOH going to accumulate. Therefore, drug loading efficiency (% EE) will be reduced. % EE of CUR and DOX when loaded separately on GO-COOH, is 79.8% and 90.4% while in the same condition, is 81.2% and 91.8% respectively.

### Release assay

**Figure 2:**
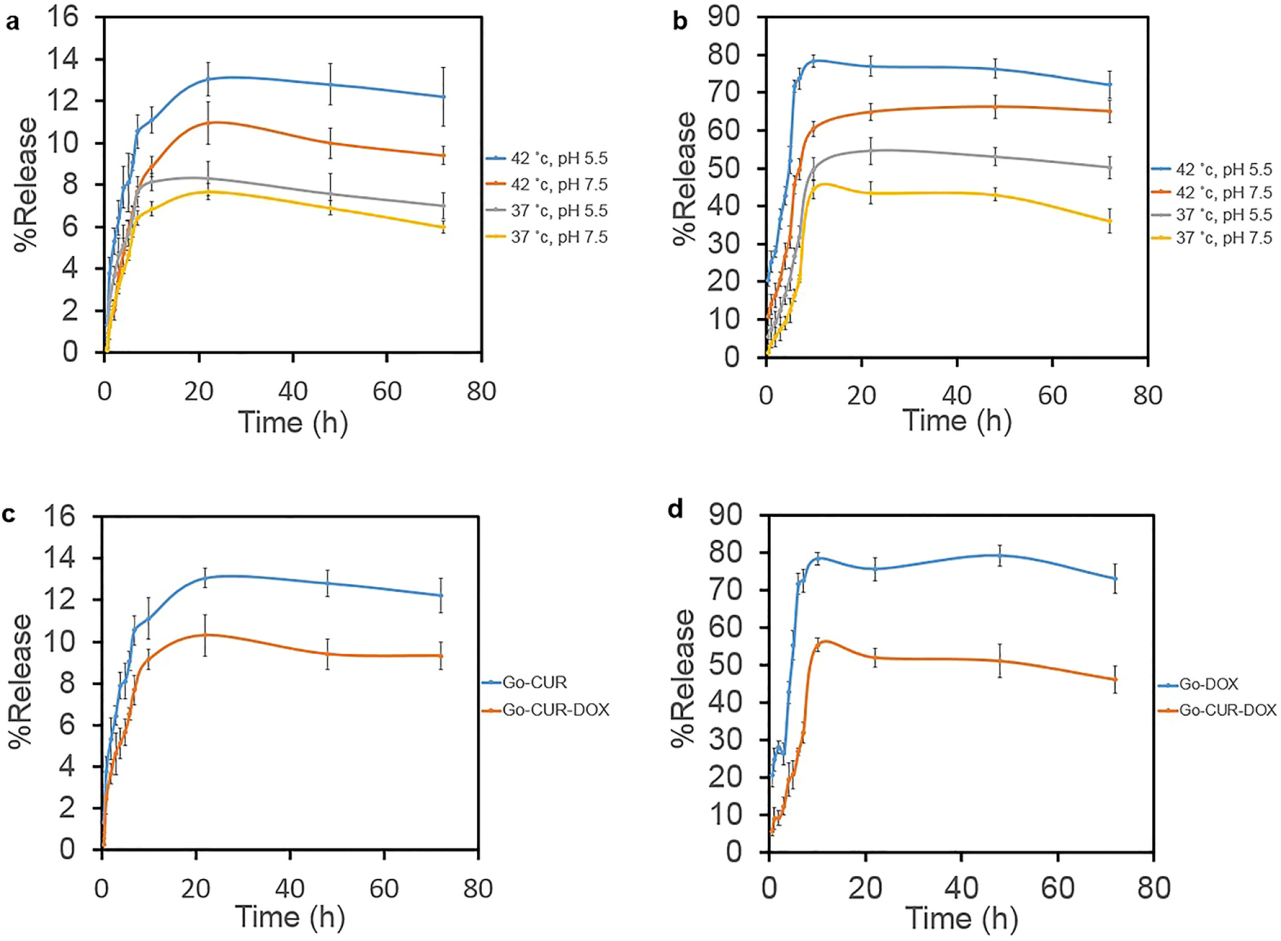
The drug release profile of (**a**) CUR, (**b**) DOX and (**c**) CUR from GO-CUR and GO-CUR-DOX nanocarriers at 37 °C and pH 7.5 (**d**) DOX from GO-DOX and GO-CUR-DOX nanocarriers at 37 °C and pH 7.5.

The choice of drug concentration in the MTT test was actually based on the IC 50 level announced in previous researches^44,45^. The IC50 dose of DOX was approximately 3 μM, so to receive the IC50 of DOX, we tested a few higher and a few lower concentrations than 3. The IC50 of CUR is about 12 times more than of DOX, so because the maximum concentration of DOX is 10, it should be mixed with 120 μM CUR. Therefore, the total concentration of DOX and CUR is 130 μM. Our system was optimized based on both loading and release. Generally, cancer cells have lower pH and higher temperature than normal cells^46^. In the in-vitro conditions, some factors impact on the rate of drug release, including pH and temperature of buffer of surrounding the nanoparticles and the structure of the GO-COOH membrane. In the current study, pH 7.5 and temperature 37 °C were considered as a physiological condition, and pH 5.5 and temperature 42 °C for cancerous cells. The result shows that the designed GO-COOH has thermo-and pH-sensitive effects leading to better delivery of anticancer drugs into tumor cells, which in turn, diminishes the side effects on normal cells. Figure 2 illustrates that CUR and DOX have the highest release rates (13.06%, and 80.23%) at pH 5.5 and temperature 42 °C, respectively. However, the lowest release rates belong to physiological conditions (7.69% for CUR and 44.5% for DOX). As Fig. 2c,d shows the release of CUR and DOX, decrease in the combination form, rather than the single form occurs. The obtained result is similar to the results of studies conducted by Malekmohammadi et al., Omidi et al. and Pourjavadi et al.^37,47,48^. In fact, high temperatures weaken the π → π* bonds and in low pH, protonation of the amine groups (-NH_2_) of DOX results in the partial dissociation of hydrogen-bonding interaction^33^. Also, decreasing the pH from 7.4 to 5.4, reduces the strength of imine bonds while increases the interactions between the protonated amino groups. The low release rate of CUR (13.06%) is related to its special structure, as well as its hydrophobicity tending to remain on GO-COOH^48^.

### Cellular uptake assay

**Figure 3:**
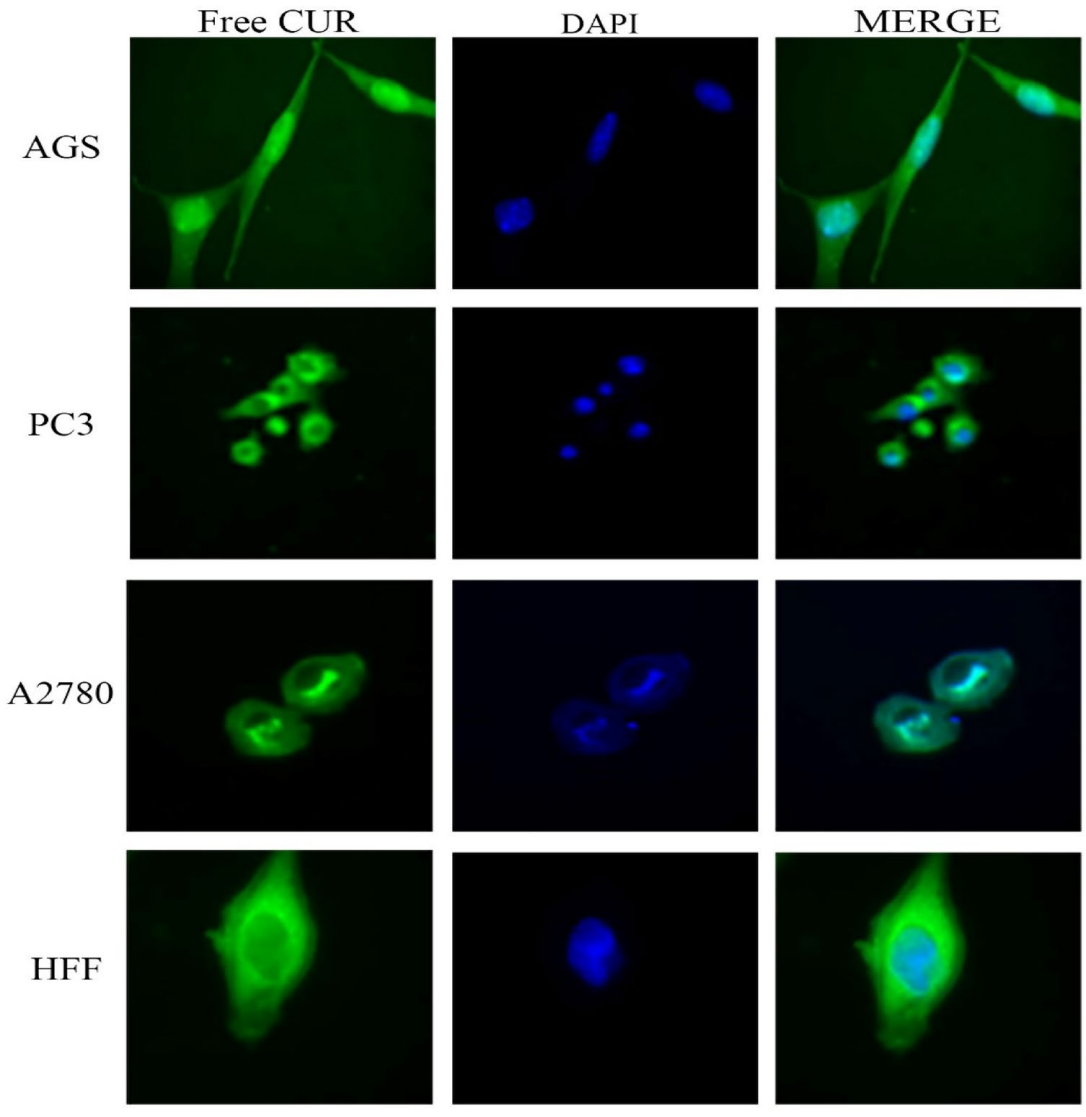
Cellular uptake images of AGS, PC3, A2780 and HFF cells incubated with free CUR for 180 min. DAPI (blue) was used for nucleus staining.

**Figure 4:**
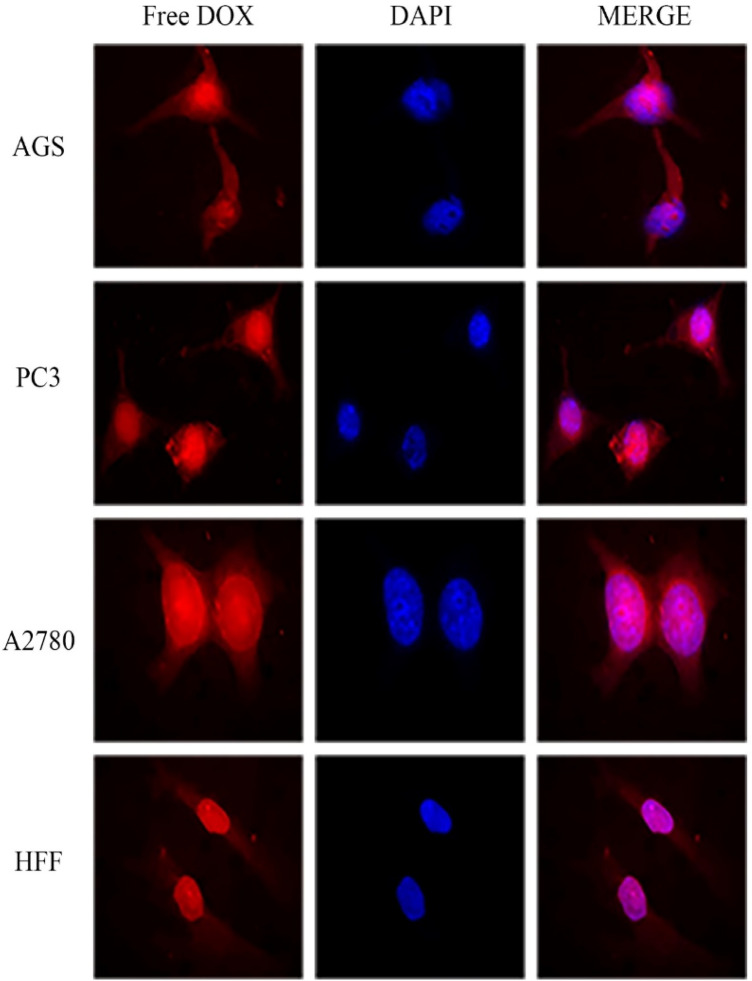
Cellular uptake images of AGS, PC3, A2780 and HFF cells incubated with free DOX for 180 min.

**Figure 5:**
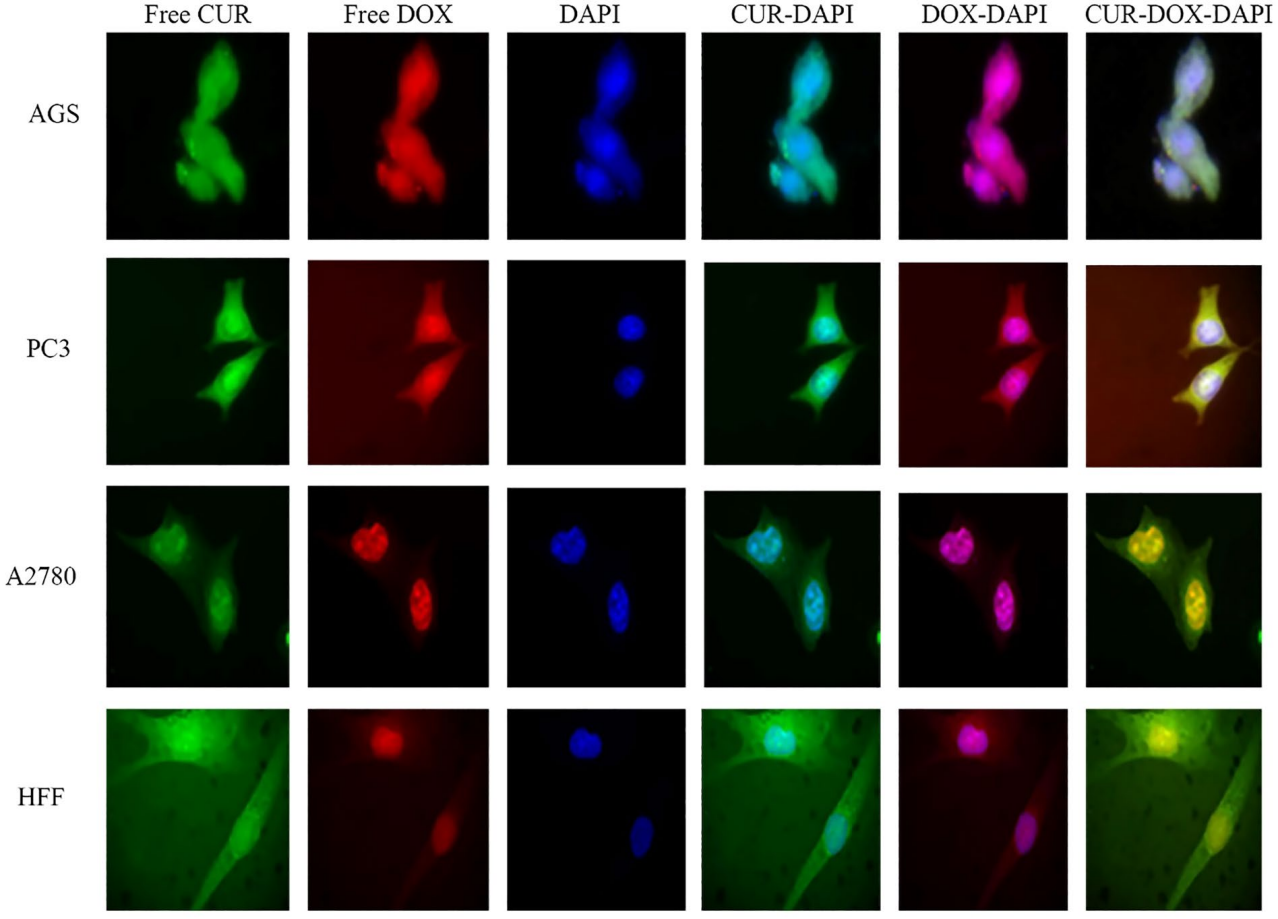
Cellular uptake images of AGS, PC3, A2780 and HFF cells incubated with FREE CUR-DOX for 180 min.

**Figure 6:**
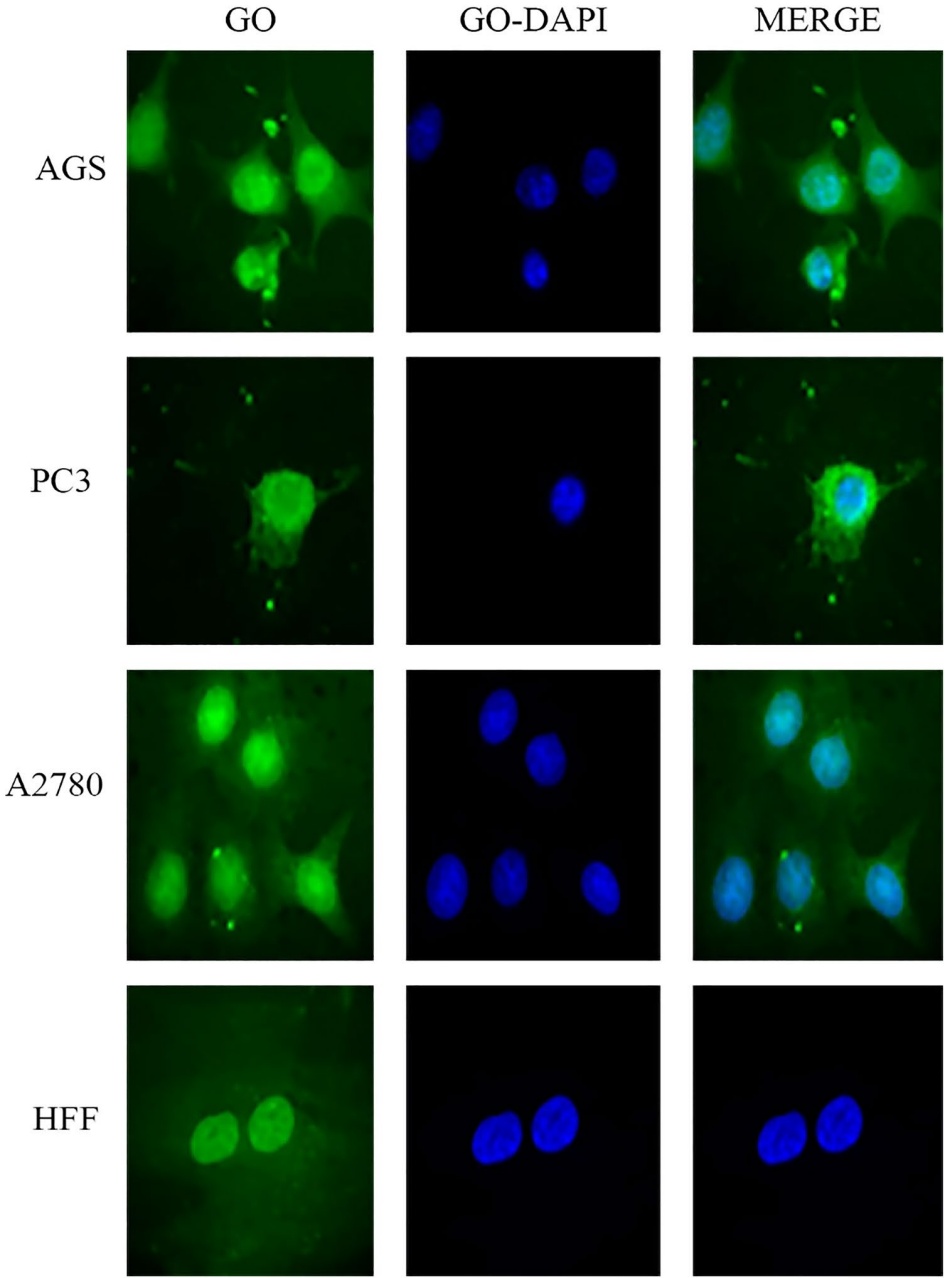
Cellular uptake images of AGS, PC3, A2780 and HFF cells incubated with GO-DIL for 180 min.

**Figure 7:**
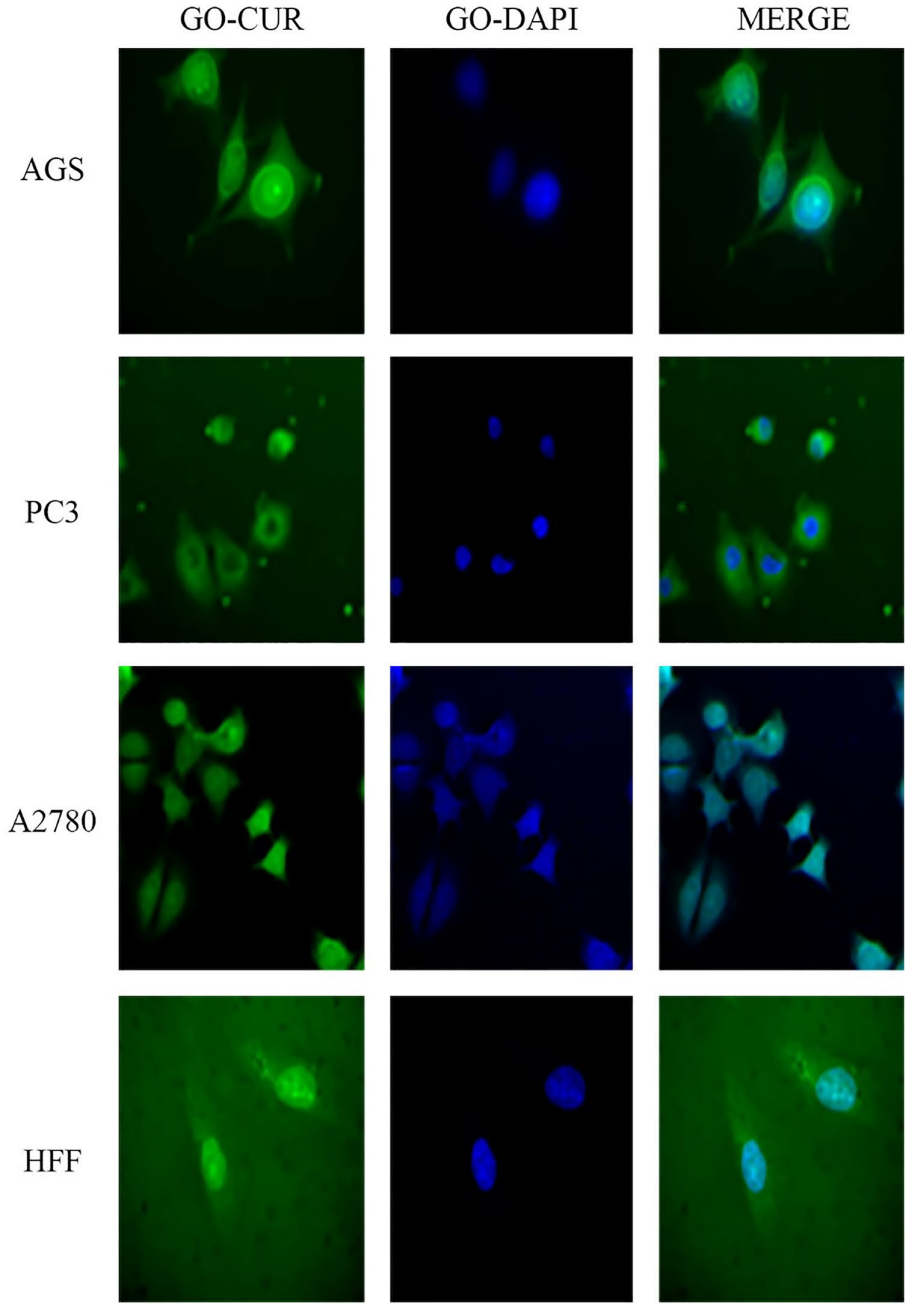
Cellular uptake images of AGS, PC3, A2780 and HFF cells incubated with GO-CUR for 180 min.

**Figure 8:**
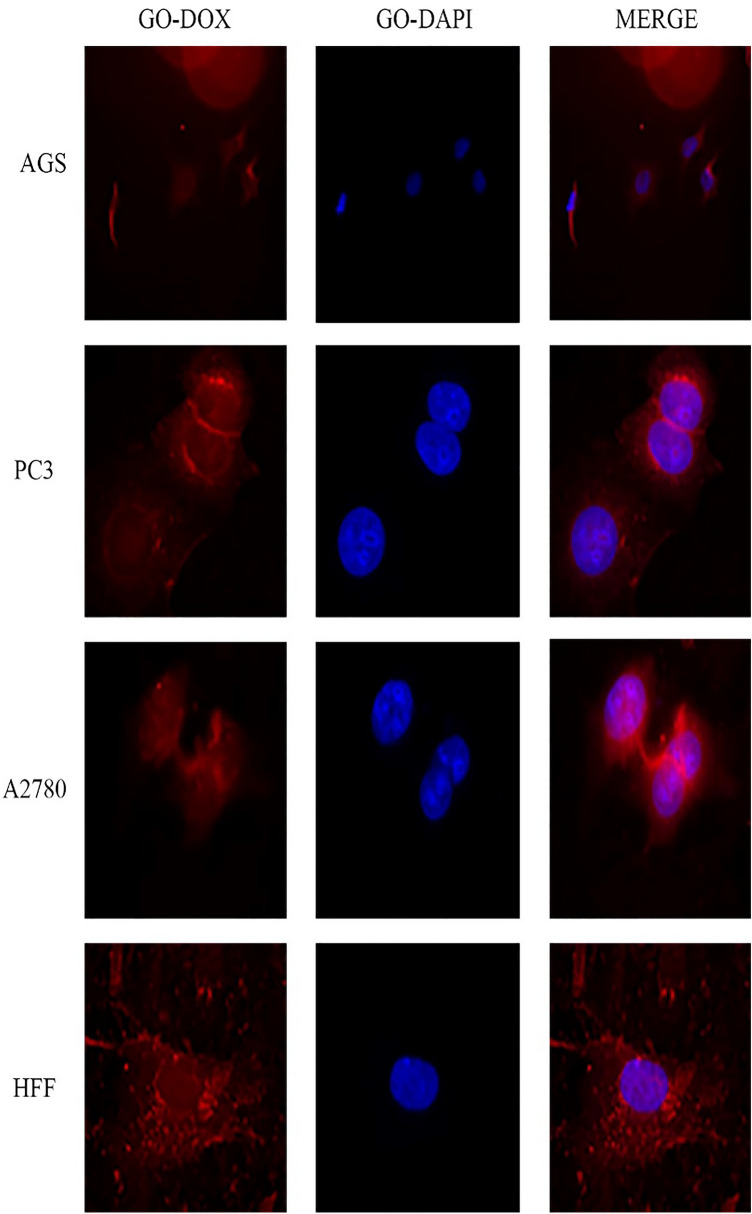
Cellular uptake images of AGS, PC3, A2780 and HFF cells incubated with GO-DOX for 180 min.

**Figure 9:**
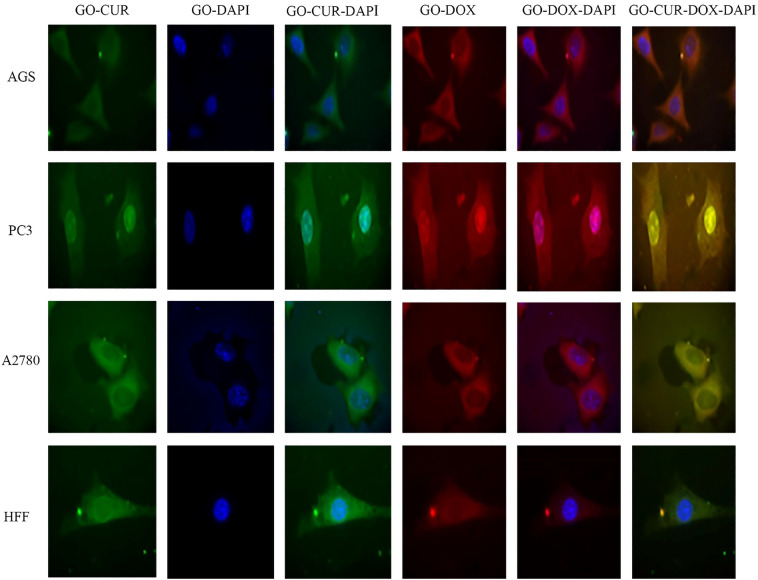
Cellular uptake images of AGS, PC3, A2780 and HFF cells incubated with GO CUR-DOX for 180 min.

Images of cellular uptake of free drugs (DOX and CUR) and loaded drugs on GO-COOH in AGS, PC3, MCF7 and HFF cell lines, which have been captured by fluorescence microscopy, were shown in Figs. 3, 4, 5, 6, 7, 8, 9. DAPI (4′,6-diamidino-2-phenylindole) dye and DIL known as DiIC18, were used for staining the nuclei of the cancer cells and GO-COOH, respectively. These figures monitor the successful transfer of DIL-labeled GO-COOH and drug-loaded GO-COOH into cells. CUR and DOX were depicted as green and red fluorescence, respectively. The intensity of fluorescence in the free form of the drug was higher than the loaded form. Some extent of free form penetrates into the cells by a diffusion mechanism through the cell membrane, the majority of them passes by endocytosis, which is the key mechanism for passing the loaded drugs. Until drugs are attached to GO-COOH, weak fluorescence will be emitted due to GO-COOH quenching property^49^. Not only the solubility of CUR as a hydrophobic drug, increases through GO-COOH, but also by the effect of Cur in the cellular internalization, it enhances the intensity of co-loaded drugs on GO-COOH compared to one drug loaded on the carrier. Also, the solubility of CUR as a hydrophobic drug, increases through GO-COOH. Captured images of HFF as a normal human cell line, show lower fluorescence than the cancerous cells which verifies the lower entry of DIL-labeled GO-COOH and drug-loaded GO-COOH into HFF cells. These results were consistent with the results of cell viability.

### Cytotoxicity assay

**Figure 10:**
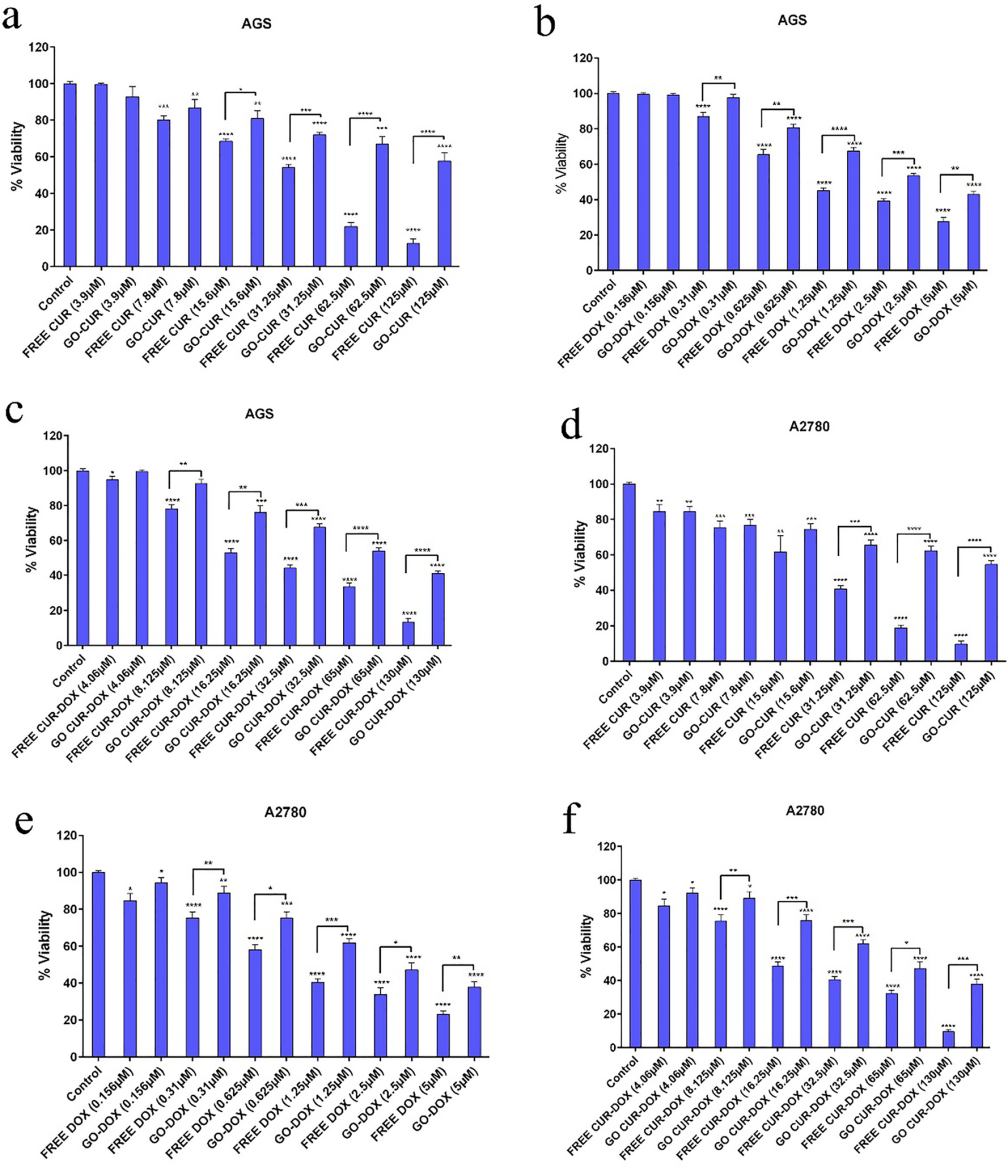
Cytotoxicity analysis of (**a**) different concentration of free CUR and GO-CUR in AGS after 48 h, (**b**) different concentration of free DOX and GO-DOX in AGS after 48 h, (**c**) different concentration of free CUR-DOX and GO-CUR-DOX in AGS after 48 h, (**d**) different concentration of free CUR and GO-CUR in A2780 after 48 h; (**e**) different concentration of free DOX and GO-DOX in A2780 after 48 h; (**f**) different concentration of free CUR-DOX and GO-CUR-DOX in A2780 after 48 h.

**Figure 11:**
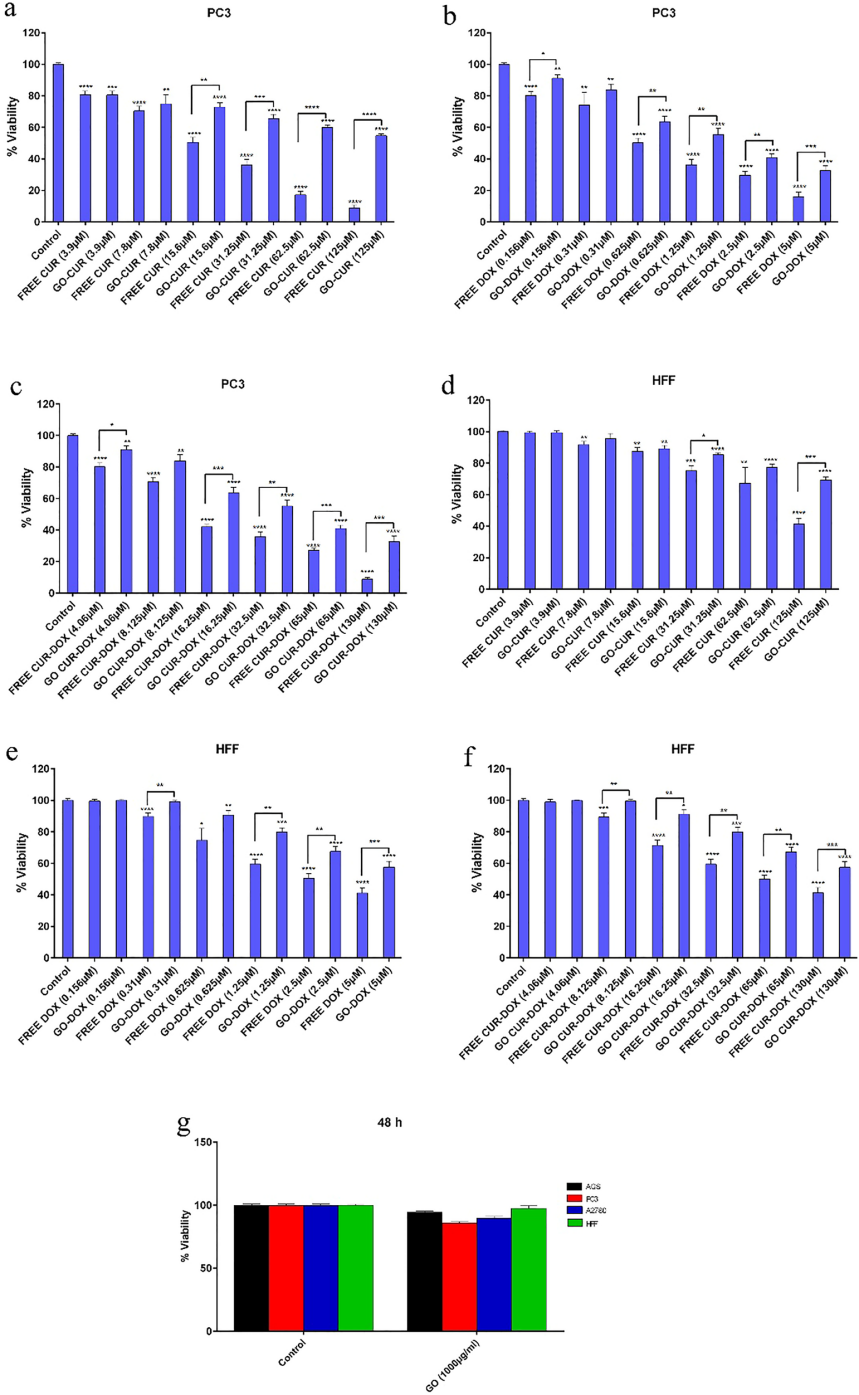
Cytotoxicity analysis of (**a**) different concentration of free CUR and GO-CUR in PC3 after 48 h; (**b**) different concentration of free DOX and GO-DOX in PC3 after 48 h; (**c**) different concentration of free CUR-DOX and GO-CUR-DOX in PC3 after 48 h; (**d**) different concentration of free CUR and GO-CUR in PC3 after 48 h; (**e**) different concentration of free DOX and GO-DOX in PC3 after 48 h; (**f**) different concentration of free CUR-DOX and GO-CUR-DOX in PC3 after 48 h; (**g**) free GO-COOH in AGS, PC3, A2780 and HFF after 48 h.

Although the use of chemotherapeutic agents is one of the main approaches for the treatment of cancer, drug resistance and its broadside effects on normal cells, have limited its efficacy. To overcome chemotherapy, cancer cells target several pathways. Therefore, combination therapy could conquer drug resistance by targeting multiple pathways in tumorigenesis. In order to achieve this goal, we loaded CUR and DOX simultaneously on GO-COOH. MTT assay was performed for evaluation of the inhibitory effects of different forms of drugs on AGS, PC3, MCF7 and HFF cell lines (Figs. 10 and 11). As can be seen in Fig. 11g, GO-COOH has little cytotoxicity. The treatment with either free form of CUR and DOX or the loaded form, has shown a dose-dependent manner in the mentioned cell lines. IC50 values of drugs are shown in Table 1. In all treatment, HFF cell as a normal cell line, had higher IC50 value than the cancer cells, resulting in less toxicity on normal cells. In the same concentration, the free forms of CUR and DOX were more toxic than the loaded form in all cell lines. Also, in all treatments, the combined form of CUR and DOX had a lower IC50 value compared to the free form of CUR-DOX. Interestingly, the current cytotoxicity assay result is inconsistent with the obtained release, in which the free form of the drug was more toxic than the form of the drug loaded on GO-COOH. Because the main goal of drug delivery with the nano-carriers, is the localized and controlled release due to lower side effects on normal cells^50^.

**Table 1:** The IC50 values of DOX and CUR on AGS, PC3, A2780 and HFF cells after 48 h.

IC50 (µM)	CUR	DOX	CUR-DOX	GO-CUR	GO-DOX	GO-CUR-DOX
AGS	45.17 ± 3.99	0.63 ± 0.99	16.52 ± 2.80	88.50 ± 5.79	1.92 ± 0.80	43.02 ± 3.19
A2780	31.96 ± 3.60	0.71 ± 0.43	9.18 ± 2.14	83.48 ± 6.10	1.55 ± 0.45	34.14 ± 2.78
PC3	20.8 ± 1.84	0.51 ± 0.41	7.17 ± 1.92	63.50 ± 4.88	0.84 ± 0.37	28.49 ± 3.26
HFF	97.40 ± 6.20	1.95 ± 1.17	44.62 ± 3.90	106.63 ± 8.42	2.93 ± 1.58	60.19 ± 6.83

### Real time PCR

**Figure 12:**
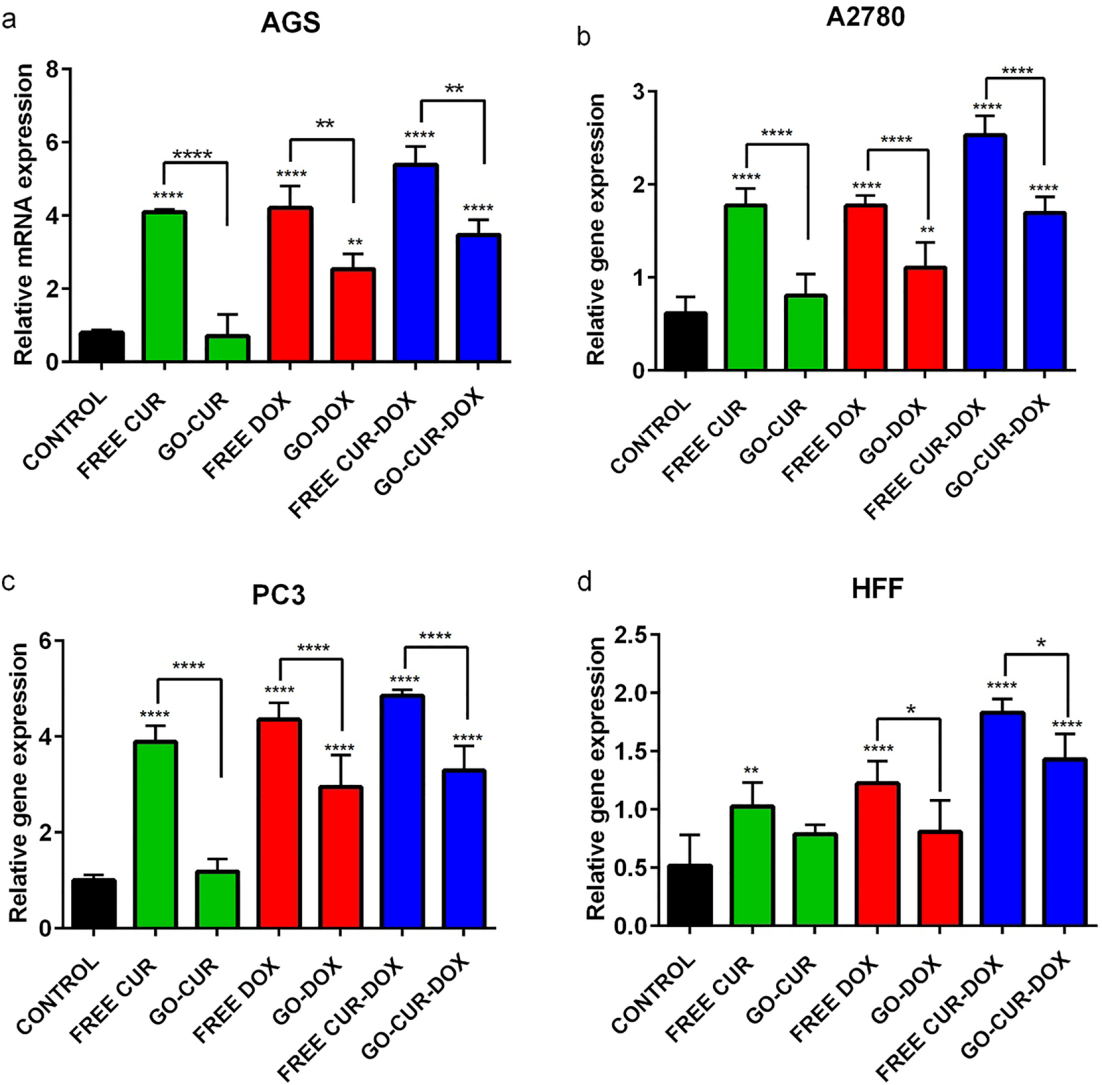
Effect of free CUR, GO-CUR, free DOX, GO-DOX, free CUR-DOX and GO-CUR-DOX on the expression of RB1 in AGS, PC3, A2780 and HFF after 48 h. Results are expressed as the mean ± standard deviation (*p < 0.05) (**p < 0.001) (****p < 0.0001).

**Figure 13:**
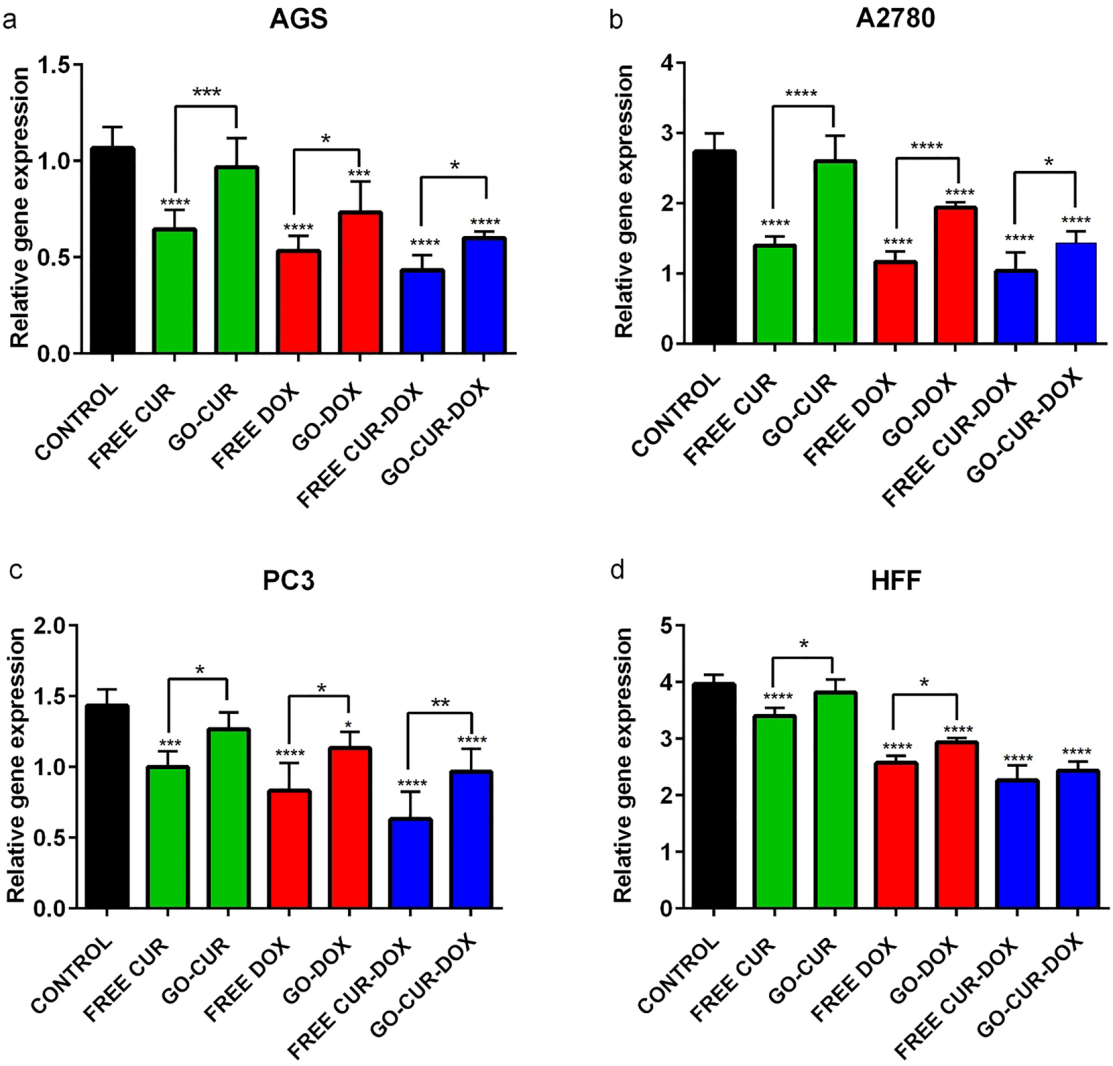
Effect of free CUR, GO-CUR, free DOX, GO-DOX, free CUR-DOX and GO-CUR-DOX on the expression of CDK2 in AGS, PC3, A2780 and HFF after 48 h. Results are expressed as the mean ± standard deviation (*p < 0.05) (**p < 0.001) (****p < 0.0001).

Expression of RB1 and CDK2 genes involved in cell cycle regulation has been studied. As shown in Figs. 12 and 13, both CUR and DOX diminished the expression of CDK2 while heightened the expression of RB1 in all treated cells. However, the amount of this effect was different between various cell lines. The expression of RB1 and CDK2 was most impacted on cancerous cell lines (AGS, PC3 and A2780) while CUR and DOX had the lowest effect on the expression of RB1 in HFF normal cell line. The earlier study was inconsistent with our result that normal cells, telomerase negative human lung fibroblast cells, displayed higher IC50 alongside more tolerance to the cytotoxic effects of CUR^51^. Free CUR and free DOX have shown more effect rather than GO-loaded drug. Also, concomitant of DOX with CUR, increased its efficacy. Increased level of RB1 beside reduced level of CDK2, suggested the arrest of cells at G1 phase of the cell cycle. Also, conducted studies exhibited increased apoptotic cell death via CUR, resulted in up-regulation of Bax and down-regulation of Bcl2^52,53^. Additionally, CUR acts via multiple mechanism including induction of cell death via suppressing NFKB^54^, activation of death receptors^55^ and induction of ROS^56^. Also, CUR increased expression of PTEN and decreased expression of E2F1, CCNE1 and CDK2 suggesting cell cycle arrest in CUR-treated cells. In fact, correlation between the induction of cell cycle arrest and apoptosis with upregulation of CDK inhibitors (p16, p21 and p27), has been demonstrated in earlier studies^57,58^. Another study showed anti-proliferation effect of CUR on Y79 RB cells through up-regulation of Rb1 and modulation of miR-26a^59^. DOX can induce two modes of cell death by the regulation of Cdc2 and Cdk2 kinase. High dose of DOX induces apoptosis via reduction of Cdc2 and Cdk2 alongside reductions in cyclin A and cyclin B levels. On the other hand, low dose of DOX causes cell death through activation of Cdc2 and Cdk2 kinases, cyclin A, cyclin B and Cdc2 after the treatment for 1 day^60^.

The biocompatibility of GO is challenging and that of GO-COOH is more challenging in literature. Some researchers have confirmed that GO was cytotoxic in high concentration, but it was biocompatible in low concentration^61–64^. Here, we used GO in low concentration (0.5) that is biocompatible^61,62^ and the results confirmed this claim. Although some researchers reported that GO-COOH had toxicity, some researchers reported that the amount of -COOH could affect and enhance biocompatibility^32^. We used the best protocol to functionalize the GO for biomedical applications. Therefore, the functionalized GO was safe and biocompatible that the results confirmed the claim.

The use of GO loaded antitumor drugs can effectively reduce the toxic and side effects of drugs on normal cells, due to controlled and prolonged release of drugs in a period of time. Therefore, burst release is reduced that can reduce anti-tumor effects but can enhance biocompatibility and avoid side effects and toxicity on normal cells. When a drug is loaded on a nanocarrier, some drug remains free in the solution and is not attached to the carrier (encapsulation efficiency). Moreover, some drugs are not released in a short period of time that can affect anti-tumor efficiency. Here, we used functionalized GO-loaded herbal drug and DOX with high encapsulation efficiency (about 80%) and the cumulative release of drug was 80% (best formula) after 48 h. In the best condition, about 60% of the drug was released in this period of time. Therefore, it seems these results were acceptable for this situation. Other researches confirmed our results in the same condition^33,65^.

## Conclusion

In this research, co-delivery of CUR and DOX in four cell lines, including AGS, PC3, MCF7 and HFF, were studied via a suitable nanoparticle, whose special characters such as broad surface and bipolar properties allowed us to load more than one drug. Although CUR and DOX were loaded on GO-COOH with high EE%, release profile of CUR was less than 15% due to its hydrophobic structure, leading to be tightly attached. Also, release rate of the free agents is depended on pH and temperature, which differentiates normal and cancerous cells. Cytotoxicity assay in all mentioned cells, displayed that the IC50 values of the loaded drugs were higher than the free form. Consequently, high drug loading, localized delivery and controlled release were achieved which led to the fewer side effects on normal cells. Moreover, PC3 showed more sensitivity to all drugs while AGS was more resistant (between the mentioned cancerous cell lines). Likewise, the result of real time PCR was inconsistent with the other results, displaying up-regulation of RB1 and down-regulation of CDK2 implied in cell cycle arrest at G1 phase. Photothermal and photodynamic therapy can be used in future studies for increasing the release rate of CUR and DOX.

## Data Availability

The data are not publicly available due to governmental policy and privacy.
